# Practical challenges in the therapeutic management of patients with dual diagnosis - epilepsy and bipolar disorder diagnosis

**DOI:** 10.1192/j.eurpsy.2026.11182

**Published:** 2026-07-31

**Authors:** O. Vasiliu, A. M. Ciobanu, A. G. Mangalagiu, B. M. Petrescu

**Affiliations:** 1 Clinical Neurosciences Department, “Carol Davila” University of Medicine and Pharmacy; 2 Psychiatry Department, “Dr. Carol Davila” University Emergency Central Military Hospital; 3 ”Prof. Dr. Al. Obregia” Psychiatry Clinical Hospital, Bucharest, Romania

## Abstract

**Introduction:**

Common clinical features, like the episodic nature of both bipolar disorder (BD) and epilepsy, a supposed common pathogenesis through kindling phenomena, and also commonalities in pharmacological approaches, like the use of anticonvulsants, should lead to a thorough exploration of this dual diagnosis. However, the comorbidity of these two conditions raises important therapeutic and monitoring challenges that need to be acknowledged by the case managers in patients with such a dual diagnosis.

**Objectives:**

The objective of this review was to explore the practical challenges of the therapeutic management in patients with both epilepsy and BD, and the potential solutions to such challenges.

**Methods:**

A literature review was conducted in three electronic databases (PubMed, EMBASE, and Clarivate/Web of Science) using the keywords „bipolar disorder,” „epilepsy,” „anticonvulsants,” „moodstabilizers,” „tolerability,” „efficacy,” for papers published in English between January 2000 and February 2025. Both primary and secondary reports were included in the analysis.

**Results:**

Several challenges regarding the management of this comorbidity have been identified in the literature: the need for better identification of BD symptoms in the context of epilepsy; the need to identify possible common pathogenetic mechanisms; exploration of the paradoxical onset of depression during the improvement of the epileptic activity when anticonvulsant treatment is administered; delineation of the anticonvulsants who possess confirmed moodstabilizing properties; the need to formulate a plan for monitoring tolerability in patients with this dual diagnosis (biological parameters); what optimization therapies can be tried in treatment-resistant BD or treatment-refractory epilepsy, in patients with dual diagnosis.

**Conclusions:**

The BD and epilepsy dual diagnosis raises essential questions for case management from both tolerability and efficacy, while no clear guidelines have been identified for these patients. The exploration of specific aspects, such as the most efficient first-line interventions, second-line options, augmentation strategies, monitoring strategies for adverse events, monitoring of plasma concentration of anticonvulsants, and the correlation of such levels with the clinical efficacy and tolerability, all of these are unmet needs for the case management of BD-epilepsy.

**Disclosure of Interest:**

None Declared

